# Exploratory Room-Level Acoustic Soundscape Monitoring of Cough-like Events Under Standard and Ventilation-Restricted Pig-Housing Conditions Using Audio Spectrogram Transformer

**DOI:** 10.3390/ani16142275

**Published:** 2026-07-22

**Authors:** Md Sharifuzzaman, Hong-Seok Mun, Md Kamrul Hasan, Jin-Gu Kang, Eddiemar B. Lagua, Hae-Rang Park, Keiven Mark B. Ampode, Young-Hwa Kim, Ahsan Mehtab, Chul-Ju Yang

**Affiliations:** 1 Animal Nutrition and Feed Science Laboratory, Department of Animal Science and Technology, Sunchon National University, Suncheon 57922, Republic of Korea; baushossain@gmail.com (M.S.); keivenmark.ampode@wpu.edu.ph (K.M.B.A.);; 2 Department of Animal Science and Veterinary Medicine, Gopalganj Science and Technology University, Gopalganj 8105, Bangladesh; 3 Department of Multimedia Engineering, Sunchon National University, Suncheon 57922, Republic of Korea; 4 Department of Poultry Science, Sylhet Agricultural University, Sylhet 3100, Bangladesh; 5 Interdisciplinary Program in IT-Bio Convergence System (BK21 Plus), Sunchon National University, Suncheon 57922, Republic of Korea; 6 College of Agriculture, Forestry, and Environmental Sciences, Western Philippines University, Aborlan 5302, Palawan, Philippines; 7 Interdisciplinary Program in IT-Bio Convergence System (BK21 Plus), Chonnam National University, Gwangju 61186, Republic of Korea

**Keywords:** precision livestock farming, room-level soundscape, cough-like events, ventilation restriction, audio spectrogram transformer, calibration, early warning, pig welfare

## Abstract

Respiratory sound monitoring is a promising non-invasive tool for precision pig farming, but practical evidence from calibrated room-level deployment under degraded air-quality conditions remains limited. This study reports a 28-day exploratory room-level case study in which 52 growing pigs were housed in two rooms: one standard-ventilation room and one ventilation-restricted room, and monitored with one microphone per room emphasizing mixed room-level soundscape monitoring rather than individual pig cough counts or replicated treatment inference. Because the design lacked independent room-level replication, all room contrasts and *p*-values were interpreted as exploratory descriptive screening summaries rather than causal treatment effects. Airflow verification, playback calibration at multiple pen positions, and background-noise spectral analysis were performed to address measurement bias. Signal inspection showed that biologically relevant vocal energy was retained after 16 kHz resampling, while class imbalance was handled by inverse-frequency weighting and macro-F1-based model selection. The Audio Spectrogram Transformer (AST) pipeline was subjected to five-fold group-blocked cross-validation, and temporal validation. The model achieved a test macro-F1 of 0.937, five-fold macro-F1 of 0.928 ± 0.019, and three-day deployment validation macro-F1 of 0.914. In this two-room dataset, the ventilation-restricted room displayed higher room-level cough-like detections, aggressive vocalizations, normal vocalizations, lower silence, reduced growth, and poorer air quality. Cough-like detections showed recurring clock-time clustering, with the most sustained elevation during 19:00–22:00 and a smaller peak around 10:00 with the highest occurrences at 20.00 (2.84 room-level cough-like detections standardized to group size). Audio-only early-warning analysis flagged deteriorated air-quality windows with AUROC = 0.91 and AUPRC = 0.88 and provided a median 34 min lead time before environmental threshold exceedance, highlighting practical utility as an early inspection cue for farmers before air-quality deterioration becomes more pronounced. Cough-like events descriptively co-varied positively with NH_3_, temperature, and CO_2_. Overall, calibrated AST-based monitoring can summarize group-level acoustic changes associated with degraded room environments, while multi-room and multi-farm replication remains necessary for causal inference and generalization.

## Simple Summary

Poor air quality in pig houses can reduce welfare and growth, but signs of respiratory discomfort are difficult to observe continuously. This study tested whether room-level sound monitoring could provide a simple warning signal. Fifty-two growing pigs were kept for 28 days in two rooms, one with standard ventilation and one with restricted ventilation. A centrally placed microphone in each room recorded pig-house sounds, and a computer model classified cough-like sounds, sneezing, aggressive vocalizations, normal vocalizations, and background silence. The restricted-ventilation room had poorer air quality, lower growth, more cough-like detections, more aggressive and normal vocalizations, and less silence than the standard room. Cough-like activity was highest in the evening, especially between 19:00 and 22:00, and it increased together with poorer air-quality indicators. These results suggest that calibrated sound monitoring may help farmers notice early changes in pig-house conditions and support welfare-oriented ventilation management. The audio-only early-warning model provided a median 34 min lead time before environmental threshold exceedance, which could give farmers a practical window to check sensors, inspect fans, and adjust ventilation before further deterioration. However, the findings are exploratory because the study used only two rooms and did not diagnose individual pigs.

## 1. Introduction

Respiratory disease is a major economic and welfare issue in pig production because it substantially reduces growth performance, feed efficiency, and carcass quality, leading to significant financial losses for farms. Diseases within the porcine respiratory disease complex (PRDC), including porcine reproductive and respiratory syndrome virus (PRRSV) and *Mycoplasma hyopneumoniae*, cause clinical symptoms such as fever, coughing, lethargy, and dyspnea that compromise animal welfare and productivity [1,2]. Economic consequences include reduced average daily gain, delayed marketing age, increased medication costs, and lower carcass weight and meat yield, with reported losses reaching hundreds of euros per pig or sow annually [1,3,4]. PRRSV alone is estimated to cause billions of dollars in annual losses in major swine-producing countries such as the United States and China by affecting growth and reproductive performance across all production stages [5,6,7]. Because routine detection often relies on visual observation and subjective clinical judgment, respiratory problems may be recognized only after substantial health and performance losses have occurred [8,9,10,11,12,13].

Among non-invasive monitoring approaches, bioacoustics is especially relevant because coughing is one of the most informative and observable respiratory signs in pigs [8,14,15]. Pig-specific studies have already established that cough sounds can be used for disease-oriented acoustic screening [14,16]. Subsequent machine-learning and deep-learning studies reported strong performance for automated pig cough detection, including continuous detection under noisy pig-house conditions [17,18,19,20]. More recent work has further extended pig cough monitoring through audio–visual systems for cough localization and 24/7 AI-based surveillance platforms evaluated under field conditions or in combination with diagnostic sampling [21,22,23]. Thus, the feasibility of automated cough detection in pigs is well supported; the main question is how to move from clip-level cough recognition toward more operational, context-aware respiratory surveillance. In the present study, however, model outputs are deliberately described as cough-like acoustic events because no event-level clinical diagnosis, pathogen confirmation, or individual-animal localization was performed during deployment.

Most pig respiratory bioacoustic studies still focus primarily on binary cough detection, disease classification, or model benchmarking, rather than on the broader temporal and environmental context in which cough-like events occur [17,18,19,20,21,24]. Comparatively less attention has been given to multiclass acoustic monitoring of the pig-house soundscape, to group-level hourly dynamics of cough-related events, and to the possibility that cough-like detections may show recurring within-day surveillance windows during continuous monitoring. Likewise, although environmental stressors such as ammonia, carbon dioxide, temperature, humidity, and dust are known to influence respiratory physiology and welfare in pigs [25,26,27], relatively few studies have examined how AI-detected cough-like events co-vary with these variables over time under practical housing conditions.

This gap is biologically important because respiratory sound production is not determined by infection status alone. Elevated ammonia and particulate exposure can damage the respiratory epithelium, impair mucociliary clearance, and increase airway irritation, while poor ventilation and heat load can further aggravate respiratory stress and alter behavior [28,29,30,31]. These mechanisms support the expectation that degraded housing conditions may be accompanied by systematic changes in room-level acoustic activity, particularly coughing-related detections. Fewer studies evaluate how a multiclass pig-house soundscape changes over time in relation to air-quality deterioration under practical housing conditions.

Accordingly, the novelty of this study lies in integrating a multiclass pig-sound recognition framework with continuous barn monitoring to quantify group-level hourly acoustic dynamics, identify recurring clock-time surveillance windows of elevated cough-like acoustic descriptors, and examine whether AI-detected cough-like events co-varied with degraded air-quality indicators. Rather than proposing a completely new cough-detection concept, this work extends existing pig bioacoustic monitoring toward a more operational, context-aware surveillance approach. Because the design compares two housing rooms over a 28-day period, the study evaluates whether a calibrated single-microphone AST pipeline can provide room-level indices of mixed acoustic activity under two contrasting room environments. Therefore, all statistical outputs are interpreted as exploratory room-level contrasts and co-variation patterns, not definitive causal effects of ventilation restriction. The objectives were therefore to: (i) develop and validate a multiclass AST-based pig-sound classifier; (ii) quantify calibrated room-level acoustic activity under standard and ventilation-restricted environments; (iii) identify recurring clock-time windows of elevated cough-like detections while accounting for routine management schedules; (iv) evaluate exploratory associations between soundscape indices, environmental variables, and growth performance; and (v) test whether audio-derived features alone can flag deteriorated air-quality windows.

## 2. Materials and Methods

### 2.1. Experimental Setup, Animal Allocation, and Ventilation Verification

A total of 52 growing crossbred pigs [(Large White × Landrace) × Duroc], including 26 gilts and 26 barrows, approximately 9 weeks of age with an average initial body weight (BW) of 29.15 kg, were used in this study. The experiment was conducted at the experimental pig farm of Sunchon National University, Republic of Korea, in June 2024.

Pigs were allocated by sex- and body-weight-stratified randomization. Within each sex, pigs were ordered by initial BW and assigned to balanced blocks; a computer-generated random sequence then assigned pigs from each block to the standard-ventilation or ventilation-restricted room. This procedure produced 13 gilts and 13 barrows per room and comparable initial BW distributions (standard: 28.80 ± 1.06 kg; restricted: 29.50 ± 1.09 kg).

Each room contained four pens. Pens 1 and 4 housed six pigs each, whereas pens 2 and 3 housed seven pigs each. Pen dimensions were 2.35 m × 2.90 m (6.815 m^2^), resulting in stocking densities of 0.88–1.03 pigs/m^2^. All pens used the same automatic wet–dry feeding system (LFS-120, IONTECH Co., Ltd., Seoul, Republic of Korea), diet, water access, lighting schedule, and caretaker routine to reduce management-related confounding.

Ventilation settings in both rooms were managed using an IONTEC smartfarm MultiController (model IFC-370, IONTEC Co., Ltd., Incheon, Republic of Korea). In the standard-ventilation room, the nominal fan-controller output was set to 80%, whereas in the ventilation-restricted room the nominal fan-controller output was set to 20%. Actual airflow at experimental fan speed rates was verified afterwards using repeated hot-wire anemometer measurements at exhaust and inlet locations during representative morning, afternoon, evening, and night periods for three days. The airflow audit confirmed a consistent operational contrast between rooms. For animal-welfare and safety reasons, the restricted-room fan output was temporarily increased for several hours whenever room ammonia concentration approached 25 ppm. These interventions were recorded and included as management covariates in time-window analyses. Because only one room represented each condition, room was treated as the environmental-exposure unit and the study was interpreted as a two-room longitudinal case study rather than a replicated treatment trial. Accordingly, room identity and ventilation condition cannot be statistically separated as independent replicated factors in this design.

**Figure 1:**
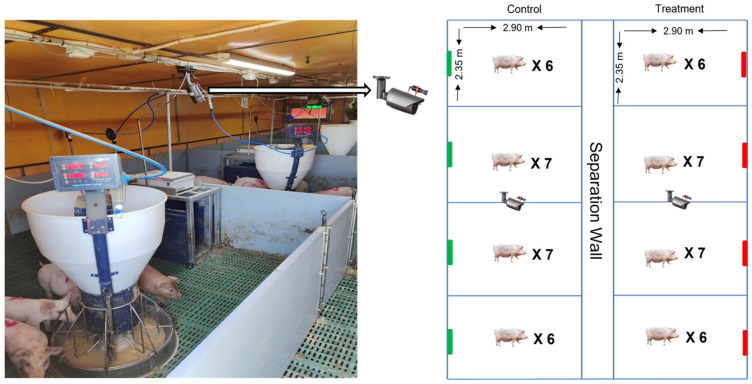
Experimental setup and room-level acoustic monitoring system. The (**left**) panel shows the overhead microphone used for continuous recording. The (**right**) panel summarizes room layout, pen distribution, and central microphone placement. The figure represents room-level mixed-sound monitoring rather than individual-animal localization.

Acoustic data were collected using one centrally suspended RØDE VideoMicro microphone (Freedman Electronics Pty Ltd., Silverwater, Australia) per room (1.4 m above the floor). With room dimensions of 9.4 m × 3.0 m, the maximum geometric distance from the microphone to a floor-level corner was approximately 5.1 m. According to the manufacturer, the microphone is a pressure-gradient cardioid condenser microphone (frequency response 100–20 kHz; sensitivity −33 dB re 1 V/Pa; signal-to-noise ratio 75 dB SPL; equivalent noise 20 dB SPL; maximum SPL 140 dB SPL; dynamic range 120 dB SPL; 3.5 mm TRS dual-mono output; and 2–5 V plug-in power). The microphone was used with the integrated Rycote^®^ Lyre^®^ shock mount (Rycote Microphone Windshields Ltd., Ashby-de-la-Zouch, UK) and WS9 windshield. This configuration was intended to capture the room-level acoustic sound-scape across the four pens. Accordingly, acoustic detections were interpreted as room-level soundscape measurements rather than pen- or pig-specific emissions. Figure 1 summarizes the room layout, animal distribution, and sensor placement.

### 2.2. Data Acquisition, and Audio Signal Quality Inspection

Growth performance data were summarized at the pen level. Individual body weight (BW) was measured at the start of the experiment and at weekly intervals thereafter, and pen-level mean BW was used for statistical analysis. Pen-level feed intake was recorded daily, and pen-level growth variables, including body weight gain, average daily gain (ADG), and feed conversion ratio (FCR), were calculated using standard formulas.

Environmental data were recorded using a Farm Note system (NareTrends Inc., Bucheon, Republic of Korea). Sensors recorded ammonia (NH_3_, ppm), carbon dioxide (CO_2_, ppm), temperature (°C), and relative humidity (RH, %) at 5 min intervals. Airborne dust concentration was measured four times daily using a portable real-time aerosol monitor (DustMate, Turnkey Instruments Ltd., Northwich, UK). Respiration rate was measured by visual observation of flank movements in four pigs per pen at 06:00, 12:00, 18:00, and 00:00. To minimize disturbance, observations were conducted from a distance without restraining the animals and under consistent environmental conditions. Respiration rate was averaged within room and time point, and daily room-level means were used for subsequent statistical analysis.

Audio tracks were extracted from stored video recordings and initially stored as MP3 files (124 kbps, stereo, 44.1 kHz). Because MP3 compression can introduce artifacts, waveform inspection, spectrogram inspection, and frequency-energy auditing were performed. The sampling-rate and bitrate choice was considered acceptable for the present room-level classification objective for three reasons. First, previous pig-cough studies indicate that diagnostically relevant cough energy is concentrated mainly in low-to-mid frequency ranges, including reported peak frequencies around 0.6–1.6 kHz [16], cough-monitoring bands around 0.5–6 kHz [8], and field pig-cough energy generally within approximately 2.5–8 kHz [20]. Second, 16 kHz resampling preserves frequencies up to 8 kHz according to the Nyquist criterion, which covered the relevant frequency range identified in both the literature [32] and our spectral audit. Third, the MP3 archive was encoded at 124 kbps stereo and 44.1 kHz, close to the widely used 128 kbps/44.1 kHz MPEG Layer III setting for low-bitrate wideband audio [33,34]. A parallel 6 h audit against lossless WAV recording further supported the suitability of the archive for room-level temporal monitoring, although lossless audio remains preferable for future fine-scale acoustic analysis. Across 600 manually reviewed cough-like, sneeze, aggressive, normal, and silence windows, most biologically relevant energy was below 7.2 kHz and no class-defining spectral component was consistently observed above 8 kHz. Resampling to 16 kHz therefore retained the relevant 0–8 kHz band while reducing computational cost and matching the AST preprocessing pipeline.

A 6 h parallel audit using lossless WAV recording was also performed. Hourly class counts derived from the MP3-based pipeline and the parallel lossless pipeline were highly concordant (overall r = 0.96; cough-like r = 0.94), indicating that the existing MP3 archive was acceptable for room-level temporal monitoring in this specific dataset. Nevertheless, MP3 storage remains a limitation for fine-scale acoustic analysis and future studies should record raw lossless audio from the outset.

The sound-class definitions and quantitative descriptors used during manual annotation and audit are presented in Table 1.

### 2.3. Acoustic Calibration, Distance Attenuation, and Background-Noise Spectral Audit

The single microphone captured mixed room-level sound rather than pen- or pig-specific emissions. To address spatial-sampling concerns, playback calibration was added using representative cough-like, sneeze, normal, and aggressive sounds played from fixed points across each room, including near-field positions, mid-pen positions, feeder-side positions, and the farthest floor-level corners. Sound pressure level and model recall were recorded by position.

Calibration confirmed the expected distance-related attenuation, but detection of standardized cough-like events remained acceptable across the room when probability thresholding and noise normalization were applied. At the farthest corner distance of approximately 5.1 m, cough-like event recall remained 0.88 in the standard room and 0.85 in the restricted room, supporting the use of room-level indices while confirming that the system should not be interpreted as an individual cough counter.

A background-noise spectral audit was performed using manually selected no-vocalization windows. The restricted room had stronger low-frequency ventilation and airflow-related energy, but the AST cough-like predictions were not explained by the noise profile alone. A noise-only classifier could distinguish room identity, yet cough-like detection remained stable after excluding high-noise windows, applying background-noise matching, and validating on room-balanced folds. The acoustic calibration and background-noise validation results are presented in Table 2.

### 2.4. AST Architecture, Cross-Validation, and Class Imbalance Management

The AST model was trained on manually annotated audio segments collected before the present 28-day monitoring study from the same university facility. The training clips came from growing pigs weighing approximately 28–80 kg that were recorded during several separate experiments between February and June 2023. Only audio from contemporaneous control animals was used, so the training corpus included recordings from both room 1 and room 2 under non-manipulated conditions. Each source experiment had its own institutional ethical approval. Because the source pigs were drawn from multiple studies, the training corpus should be viewed as a facility-specific pig-sound dataset spanning a grower-weight range rather than as a clinically characterized respiratory-disease dataset.

Annotation was performed in Label Studio (v1.12) using synchronized audio playback and waveform inspection. Annotators were trained on representative examples before formal labeling, and ambiguous clips were escalated for secondary review. Class definitions and reference examples were reviewed by an expert veterinarian and an experienced farm manager to maintain biological relevance and practical consistency. Because both training and deployment data originated from the same facility, model performance is reported as internal site-specific validation rather than external generalization.

A total of 8094 sound segments were labeled and categorized into five acoustic classes: cough-like events (*n* = 2995), sneezing (*n* = 1414), aggressive vocalizations (*n* = 879), normal vocalizations (*n* = 1143), and silence (*n* = 1663). To assess annotation reliability, a randomly selected subset of 1600 audio segments (~20% of the labeled dataset) was independently annotated by a second trained reviewer who was blinded to the initial labels. Inter-annotator agreement was quantified using Cohen’s kappa, yielding an overall agreement of κ = 0.82, indicating substantial agreement between annotators. For discrepant cases, the two annotators jointly reviewed the corresponding waveform and audio playback, and the final label was assigned by consensus. This procedure was used to refine the annotation protocol and reduce subjective labeling drift before final model training.

For model development, a pretrained Audio Spectrogram Transformer (MIT/ast-finetuned-audioset-10-10-0.4593) was fine-tuned for five-class pig-sound classification. The labeled dataset was divided into training (70%), validation (10%), and test (20%) subsets using stratified random sampling. The AST follows the vision-transformer concept applied to audio: the waveform is converted to a log-mel spectrogram, the spectrogram is divided into fixed-size time-frequency patches, each patch is linearly projected into an embedding vector, positional embeddings are added, and the sequence is processed through stacked multi-head self-attention encoder blocks. A classification token summarizes the sequence and is passed to a task-specific multilayer perceptron head for five-class prediction [35,36].

Clips were converted to mono, resampled to 16 kHz, normalized, and standardized to 1.0 s by trimming or zero-padding. Training augmentation included random temporal shift, gain perturbation, and additive barn-noise mixing. Validation and test clips were not augmented.

**Figure 2:**
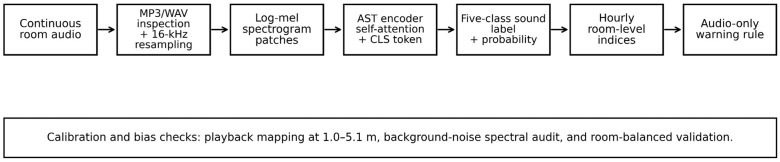
Audio Spectrogram Transformer-based monitoring workflow. The schematic shows audio preprocessing, log-mel patch generation, AST self-attention encoding, five-class prediction, hourly room-level aggregation, and audio-only warning logic. Calibration and room-balanced validation were performed to reduce spatial and background-noise bias.

To reduce leakage risk, all clips from the same parent recording and recording date were kept within the same fold. The analysis used five-fold group-blocked cross-validation in addition to the original hold-out split. Model selection was based on validation macro-F1 rather than accuracy. Class imbalance was handled through inverse-frequency class weighting, balanced mini-batch sampling, and macro-F1 reporting. The smallest class, aggressive vocalization, was monitored separately for recall stability. Hyperparameters were selected on the validation set, and the final configuration used a learning rate of 2 × 10^−5^, batch size of 8, and up to 12 epochs with early stopping. The final model and label encoder were saved for continuous-audio inference. The AST-based monitoring workflow is presented in Figure 2.

The AST was compared with five baselines: MFCC-SVM, Random Forest, XGBoost, MFCC-CNN, and Mel-spectrogram CNN. All models used the same training, validation, and test partitions, and performance was summarized using accuracy, macro-F1, weighted-F1, class-wise precision, class-wise recall, and one-vs-rest ROC-AUC.

Deployment validation was performed to three manually annotated days distributed across the monitoring period. This provided a temporal drift check across early, middle, and late study days. Manual annotators were blinded to room condition and day. Disagreements were resolved by consensus, and inter-annotator agreement on the expanded validation subset was κ = 0.84.

Manual labeling and model training were performed on a Windows 11 workstation equipped with an Intel^®^ Core™ i5-14400 CPU, 32 GB RAM, and an NVIDIA MSI GeForce RTX GPU with 6 GB dedicated memory. Continuous-audio inference and large-scale evaluation were conducted on a separate high-performance workstation equipped with an Intel^®^ Core™ i7-13700K CPU, 64 GB RAM, and an NVIDIA RTX GPU with 24 GB dedicated memory.

### 2.5. Continuous Inference and Overlapping-Event Handling

For inference, continuous audio was segmented into overlapping 1.0 s windows with a 0.6 s hop. During manual annotation, most cough-like events, sneezes, and routine vocalizations were found to be shorter than 1.0 s. Each window received one dominant-class label, so the output represents window-level room detections rather than exact counts of individual pig vocal emissions. Simultaneous events within one window could therefore be merged into a single detection, whereas prolonged aggressive episodes could appear in adjacent windows. Windows with maximum class probability < 0.7 were reassigned to silence, aligned to clock time using synchronized SMI files, and aggregated hourly into detection counts, time occupancy, and percentage occupancy. These metrics were treated as group-size-standardized room-level indices. Because the environmental contrast was applied at room level and only one room represented each condition, acoustic outputs were interpreted as descriptive room-level temporal patterns and exploratory contrasts rather than replicated treatment effects.

To assess the impact of overlapping sounds, a top-two-label audit was added to 2000 randomly selected deployment windows. Overlap or ambiguity was identified in 4.6% of windows. Reanalysis excluding ambiguous windows did not change the direction of room-level contrasts for cough-like, aggressive, normal, or silence classes, although absolute event counts decreased slightly. The manuscript therefore reports soundscape indices rather than exact event counts.

### 2.6. Acoustic Data Analysis: Group-Size-Standardized Room-Level Sound Detection and Time Occupancy

Because only one microphone and one room were available per condition, acoustic analyses summarized repeated room-level observations rather than independent pig-level measurements. Counts divided by pig number were used only as a scaling device for descriptive comparison between rooms. Consequently, model coefficients, *p*-values, and fit statistics were interpreted descriptively. Thus, these outputs should be read as hypothesis-generating summaries rather than as inferential evidence of causal ventilation effects or pig-level disease status. The acoustic output and other related data are provided as Supplementary Materials.

#### 2.6.1. Group-Normalized Sound Detection Rate Analysis

For each hour and day, detected windows were divided by the number of pigs housed in the corresponding room to obtain group-size-standardized room-level detection rates. Hourly detections were analyzed using Gamma GLMs with a log link including room condition and sound class as fixed factors, with day and hour included as temporal covariates. Daily detections were analyzed using the same Gamma-GLM framework with room condition and sound class as fixed factors and day as a covariate. Because the study used one room per condition, Wald statistics, model coefficients, *p*-values, and estimated marginal means were retained only as descriptive summaries of within-dataset room-level contrasts and temporal directionality, not as replicated treatment inference.

#### 2.6.2. Group-Normalized Sound Duration Analysis

Hourly and daily sound duration values were calculated as the summed duration of detected windows for each sound class and then standardized by the number of pigs in the room. Non-zero hourly and daily durations were analyzed using Gamma GLMs with a log link and the same explanatory structure used for detection-rate analysis. Back-transformed estimated marginal means were used to summarize the direction and magnitude of standard-ventilation versus ventilation-restricted room contrasts. Zero-duration periods were retained in occupancy analyses but were not included in Gamma duration models because Gamma models do not accommodate zero values.

#### 2.6.3. Time Occupancy Analysis

For hourly occupancy, the duration of each sound class was expressed as the percentage of the 3600 s hour occupied by that sound class. For daily occupancy, the duration of each sound class was expressed as the percentage of the 24 h day occupied by that sound class. Proportions were converted to the 0–1 scale and logit-transformed using a small constant (ε = 0.001) to accommodate zero values. Separate exploratory logit-linear models were fitted for each acoustic class to summarize room-level hourly and daily contrasts. Room condition was included as a fixed effect, whereas day and/or hour terms were used to describe repeated temporal structure. High pseudo-R^2^ or R^2^ values were interpreted cautiously as within-dataset fit summaries rather than evidence of independent causal effects.

### 2.7. Day–Night Clock-Time Sound Pattern Analysis

To summarize clock-time structure, the 24 h cycle was divided into day (06:00–17:59) and night (18:00–05:59). Negative binomial GLMs were fitted separately for each sound class because hourly detection counts were discrete and over-dispersed.

$$log\left(E\left[Y\right]\right)=\beta_{o}+\beta_{1}\left(Group\right)+\beta_{2}\left(Period\right)+\beta_{3}\left(Group\times Period\right)$$

Here, Y denotes the group-size-standardized room-level detections per hour, room condition represents the standard-ventilation versus ventilation-restricted room, and period represents day versus night. Model outputs were interpreted descriptively as room-level contrasts and day–night patterns rather than as replicated treatment effects.

### 2.8. Daily Cough-like Surveillance Windows

Surveillance-window analysis focused on cough-like events because they were treated as the most biologically relevant acoustic descriptor of possible respiratory irritation or discomfort. The dataset was filtered to cough-like event windows, and mean hourly group-size-standardized room-level detections were averaged across study days. Temporal surveillance-window models included hour of day, day, room, feeding schedule, scheduled caretaker entry, cleaning/checking periods, lighting period, and recorded emergency fan adjustments. This was done to reduce the possibility that apparent cough-like clustering was driven largely by routine management activity.

To reduce short-term noise and highlight recurring temporal structure, a 3 h centered rolling mean was applied to the hourly mean values:

$${RollingMean}_{h}=\frac{1}{3}\sum_{i=h-1}^{h+1}{\left(CountPerPig\right)}_{i}$$

Hours with the highest rolling mean values were interpreted as periods of elevated cough-like events activity and therefore as potentially informative surveillance windows rather than clinical risk thresholds. For visualization, two complementary summaries were used:

- Hourly cough-like profile: line plot of mean group-size-standardized room-level cough-like detections across 24 h with the rolling mean overlay.
- Room × hour heatmap: two-dimensional heatmap showing mean cough-like intensity across hours for the two rooms.

### 2.9. Environmental Analysis

Daily mean NH_3_, CO_2_, temperature, relative humidity, and dust values were summarized for each room to describe the environmental contrast. Because paired daily values were not normally distributed, room-level differences were summarized with the Wilcoxon signed-rank test. Relative difference, rather than a formal standardized effect size, was calculated as:

$$Effect\;size=\frac{Ventilation\;restricted\;room\;mean-Standard\;room\;mean}{Standard\;room\;mean}$$

Boxplots were used to visualize the daily distributions of each environmental variable by room, and paired line plots were used to show day-wise trajectories across the 28-day period. Because only one room represented each condition, these outputs were interpreted as descriptive summaries of environmental contrast.

### 2.10. Correlation Analysis

Exploratory pairwise Spearman correlations were computed using daily room-level summaries over the 28-day monitoring period. Variables included group-size-standardized sound detections, environmental variables, and respiration rate. Correlations were computed separately within each room condition to avoid conflating between-room differences with within-room temporal co-variation.

Because each room contributed only 28 daily observations, these correlations were interpreted as exploratory rather than confirmatory. Correlation matrices were generated independently for each room and visualized as two-panel heatmaps.

### 2.11. Statistical Analysis and Early-Warning Evaluation

The statistical framework was established to align the unit of inference with the design. Environmental exposure was assigned at room level, with one room per condition. Therefore, repeated hourly and daily observations were analyzed as longitudinal room-level time series. *p*-values from within-study models were retained only as descriptive screening statistics, while effect sizes, bootstrap confidence intervals, and directionality were emphasized. No *p*-value was interpreted as evidence for a causal ventilation-treatment effect, independent room-level replication, or confirmed clinical disease status.

Growth outcomes were analyzed at the pen level but interpreted as room-associated descriptive contrasts because pens were nested within room. Initial BW was included as a covariate, and baseline BW balance, adjusted mean differences, 95% confidence intervals, and percent differences were reported.

An audio-only early-warning model was developed to test whether deteriorated conditions could be inferred from sound alone. The target was an hourly deteriorated-environment label defined as NH_3_ ≥ 20 ppm, CO_2_ ≥ 1500 ppm, or simultaneous temperature elevation above the room-specific 75th percentile. Predictors included rolling cough-like rate, aggressive vocalization rate, normal vocalization rate, silence occupancy, soundscape entropy, and the previous-hour slope of non-silence activity. Performance was evaluated using leave-one-day-out cross-validation and summarized by AUROC, AUPRC, sensitivity, specificity, and median lead time. Median lead time was defined as the time between the first audio-only warning and the subsequent environmental threshold exceedance within the same room-level time series.

All analyses and visualizations were performed using Python (v3.11) with the pandas, matplotlib, and seaborn libraries.

## 3. Results

### 3.1. Acoustic Quality Assurance, Calibration, and Signal Interpretation

Signal inspection indicated that the five target sound classes showed distinct time-frequency structures after 16 kHz resampling. Cough-like events were characterized by abrupt broadband bursts, sneezing by shorter nasal expulsive sounds, aggressive vocalizations by longer and higher-amplitude voiced segments, and normal vocalizations by lower-intensity routine calls. Playback calibration showed that the single microphone detected standardized cough-like events across the room, but sensitivity declined with distance. The far-corner recall values of 0.88 and 0.85 indicate that some distant or masked events could still be missed. Consequently, all acoustic outcomes were retained as calibrated room-level indices rather than individual-animal counts.

Background-noise analysis confirmed that the two rooms had different acoustic noise profiles. However, model performance remained stable after noise matching and high-noise-window exclusion. This supports the interpretation that the AST did not rely solely on ventilation noise fingerprints, although residual background-noise bias cannot be fully excluded without multi-microphone localization.

### 3.2. Internal, Cross-Validation, and Temporal Model Performance

**Figure 3:**
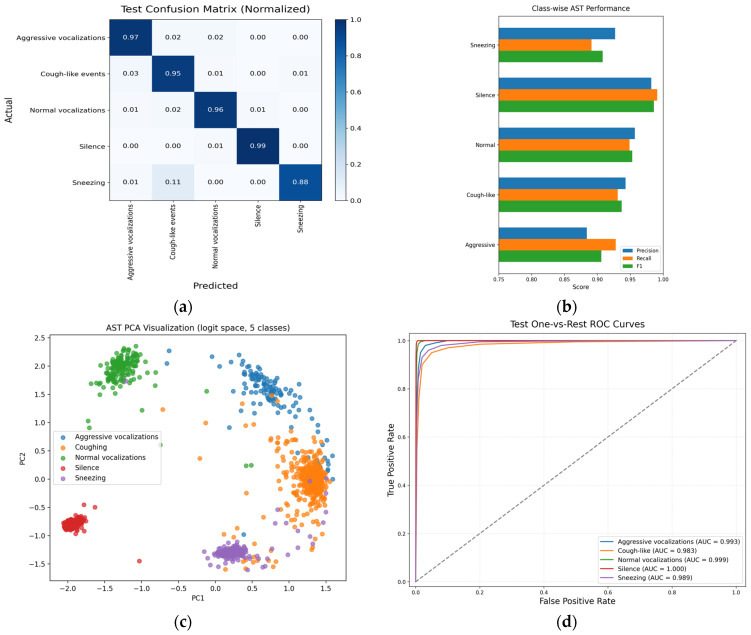
Internal performance evaluation of the AST model. (**a**) confusion matrix for the five sound categories. (**b**) class-wise precision, recall, and F1-score (**c**) PCA visualization of model output logits. (**d**) ROC curves for each class. These panels summarize within-facility model performance.

The final AST model achieved strong performance. On the group-blocked hold-out test set, accuracy was 0.948 and macro-F1 was 0.937. Five-fold group-blocked cross-validation produced a mean macro-F1 of 0.928 ± 0.019, indicating that performance was stable across blocked partitions (Table 3). Baseline comparison of all developed models is presented in Table 4. The normalized confusion matrix (Figure 3a) indicates minimal misclassification between classes. Class-wise results also addressed the minimal imbalance concern. Cough-like and silence classes were highly reliable, while aggressive vocalizations showed lower precision but acceptable recall (Figure 3b). The PCA visualization (Figure 3c) demonstrates clear separation of sound categories in the learned feature space. Furthermore, ROC analysis (Figure 3d) shows high AUC values for all classes, supporting the internal performance and feasibility of the proposed multiclass acoustic detection framework within the present facility.

The three-day deployment validation produced macro-F1 of 0.914, showing moderate temporal robustness across the 28-day monitoring period.

### 3.3. Ventilation Verification, and Environmental Contrast

Direct airflow measurement (readings were taken afterwards with the same fan speed set up) documented the operational contrast between rooms. Mean airflow was 5180 ± 380 m^3^/h in the standard room and 1420 ± 210 m^3^/h in the restricted room, corresponding to a 72.6% reduction in measured air exchange capacity during routine operation. Emergency fan increases were recorded on seven occasions when ammonia approached the safety threshold.

Environmental measurements showed markedly poorer air quality in the restricted room. NH_3_, CO_2_, temperature, and dust were higher in the restricted room, while RH differed only modestly. These differences suggest that the two-room setup produced a strong environmental contrast, but they do not establish independent replicated treatment effects. Verified airflow and environmental contrast between rooms are presented in Table 5.

**Figure 4:**
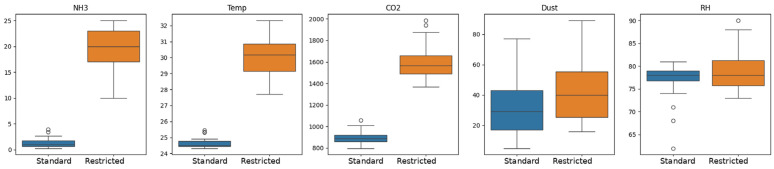
Distribution of environmental variables in the two rooms. Boxplots summarize daily values across the 28-day observation period. These values are environmental contrast between two rooms, not replicated treatment inference.

**Figure 5:**
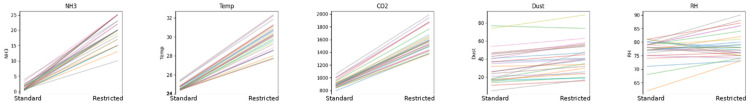
Day-wise trajectories of environmental variables in the two rooms. Paired line plots illustrate persistent separation between the standard-ventilation and ventilation-restricted rooms across the 28-day period.

Figure 4 and Figure 5 show that this environmental separation persisted across days rather than being driven by a few isolated measurements.

### 3.4. Growth Performance Under the Two Room Environments

#### 3.4.1. Weekly Growth Dynamics

Body weight increased over time in both rooms, indicating normal growth progression. In the descriptive mixed-model summary, the ventilation-restricted room showed a lower BW than the standard-ventilation room across the observation period, and the magnitude of this room-level contrast increased over time (Table 6). Because the environmental exposure was applied to one room per condition, these *p*-values are reported as descriptive screening statistics and should not be interpreted as confirmatory replicated treatment effects.

Average daily gain was lower in the ventilation-restricted room than in the standard-ventilation room in the descriptive weekly summary. The week term indicated temporal variation in ADG, whereas the room × week pattern did not suggest a strong additional increase or decrease in the room-level ADG contrast over time. These results are interpreted as room-level performance trends under the two housing environments.

Average daily feed intake was lower in the ventilation-restricted room and varied across weeks. The room × week pattern indicated that the difference in feed intake became more pronounced in later weeks. This pattern is consistent with reduced intake under a degraded room environment, but causal inference remains limited by the one-room-per-condition design.

Feed conversion ratio was less clearly separated between rooms than BW, ADG, or ADFI. FCR changed over time as pigs grew, but the room-level contrast in FCR should be interpreted cautiously because pen-level performance data were nested within two rooms only.

#### 3.4.2. Overall Growth Performance (ANCOVA-Adjusted)

Baseline BW was similar between rooms after stratified randomization. Over 28 days, pigs in the ventilation-restricted room had descriptively lower ADG and ADFI than pigs in the standard room, while FCR was less clearly separated (Table 7). Because pens were nested within one room per condition, these results are descriptive room-associated contrasts. They support consistency between poorer air quality, reduced feed intake, and lower growth in this case study, but they do not prove a population-level ventilation treatment effect.

### 3.5. Acoustic Activity: Group-Normalized Sound Detection Rates and Time Occupancy

These results represent mixed room-level acoustic activity standardized by group size, not individual event counts. The direction of the acoustic contrast was consistent after excluding high-noise windows and ambiguous overlap windows, supporting the robustness of the room-level pattern. Nevertheless, the acoustic difference could still reflect combined environmental, behavioral, management, and room-specific factors.

#### 3.5.1. Group-Normalized Sound Detection Rates

Descriptive Gamma-GLM outputs indicated higher room-level detection activity in the ventilation-restricted room than in the standard room, together with expected variation among sound classes and across day and hour. Hourly estimated marginal means were higher in the ventilation-restricted room for cough-like events (2.28 vs 1.42), sneezing (1.58 vs. 0.98), aggressive vocalizations (7.47 vs. 4.65), and normal vocalizations (6.27 vs. 3.90) (Table 8).

Daily aggregated detections showed the same direction as the hourly summaries. Estimated marginal means indicated higher group-size-standardized room-level detections in the ventilation-restricted room than in the standard-ventilation room. At the event level, aggressive and normal sounds were the most frequent, followed by cough-like events and sneezing (Table 8).

#### 3.5.2. Group-Normalized Sound Duration

An analogous pattern was observed for standardized hourly and daily sound duration. The ventilation-restricted room showed longer occupancy durations for cough-like events, sneezing, aggressive vocalizations, and normal vocalizations than the standard room (Table 9), indicating a broader increase in room-level acoustic activity across multiple sound classes.

#### 3.5.3. Time Occupancy of Sound Events

Hourly occupancy models showed the clearest room-level contrasts for cough-like events, aggressive vocalizations, normal vocalizations, and silence (Table 10). Back-transformed estimates indicated higher hourly occupancy of cough-like events (0.95% vs. 0.41%), aggressive vocalizations (3.22% vs. 1.02%), and normal vocalizations (1.83% vs. 1.18%) in the ventilation-restricted room, together with lower silence (92.48% vs. 96.10%).

Daily occupancy models again showed more time allocated to cough-like events, aggressive vocalizations, and normal vocalizations in the ventilation-restricted room and less time to silence (Table 11). These contrasts were directionally consistent with the count and duration summaries, although pseudo-R^2^ values should be interpreted cautiously because repeated observations from the same two rooms can inflate apparent model fit.

**Figure 6:**
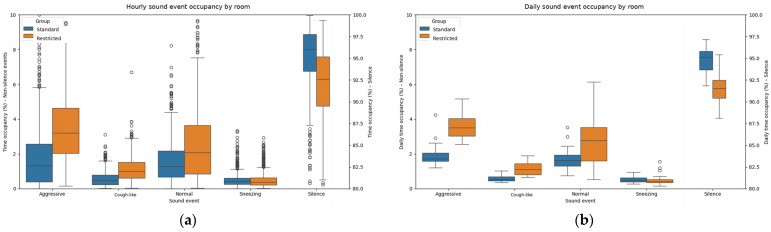
Time occupancy of sound events in the two rooms. (**a**) Hourly occupancy; (**b**) daily occupancy. Boxplots summarize the percentage of each hour/day occupied by sound classes. Values are interpreted as room-level soundscape occupancy.

Hourly and daily time occupancy of sound events in the standard-ventilation and ventilation-restricted rooms are presented in Figure 6.

Taken together, the hourly and daily summaries point to a consistent within-dataset elevation in room-level acoustic activity in the ventilation-restricted room, particularly for cough-like events and aggressive vocalizations.

### 3.6. Day–Night Clock-Time Dynamics of Sound Events

**Figure 7:**
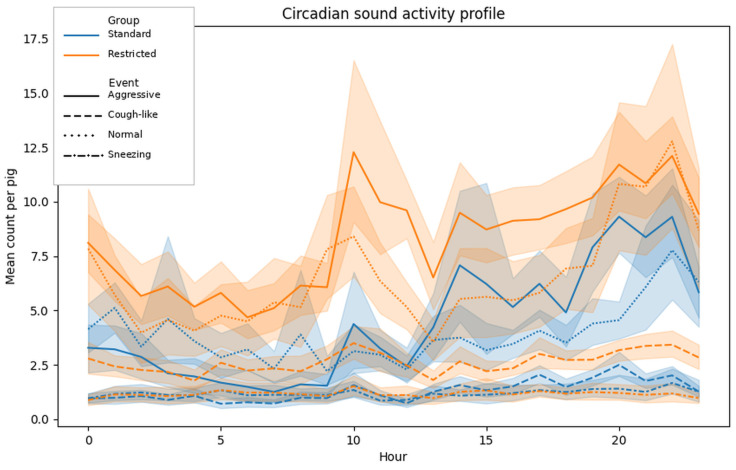
Twenty-four-hour profile of group-size-standardized room-level sound detection rates. Lines show model-derived descriptive means for aggressive, cough-like, normal, and sneezing classes.

Across the 24 h cycle, sound detections showed clear clock-time structure, with aggressive and normal vocalizations tending to increase during evening and night hours (Figure 7). The ventilation-restricted room remained acoustically more active than the standard room across most hours.

Descriptive negative binomial models suggested little day–night modulation of cough-like events and sneezing, whereas aggressive and normal vocalizations showed clearer period effects (Table 12). For cough-like events, the main contrast was between rooms rather than between day and night. For aggressive and normal vocalizations, both room condition and clock-time period contributed to the observed pattern.

**Figure 8:**
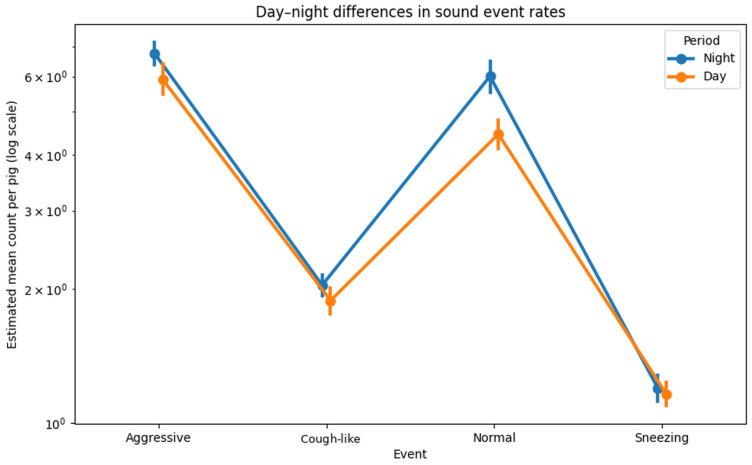
Day–night differences in group-normalized sound detection rates pooled across the two rooms. Points represent model-derived descriptive means and error bars indicate 95% confidence intervals.

To illustrate overall day–night acoustic patterns without implying replicated treatment effects, Figure 8 presents pooled day–night differences in sound event rates across both rooms.

### 3.7. Cough-like Surveillance Windows

The surveillance-window analysis revealed cough-like detections were highest during 19:00–22:00, with the largest hourly mean at 20:00, and showed a smaller secondary elevation around 10:00. The pattern persisted after management covariate (feeding, caretaker entry, lighting period, cleaning/checking periods, and emergency fan operations) adjustment, although the study cannot determine whether the timing was driven by physiology, animal activity, or room routines.

**Figure 9:**
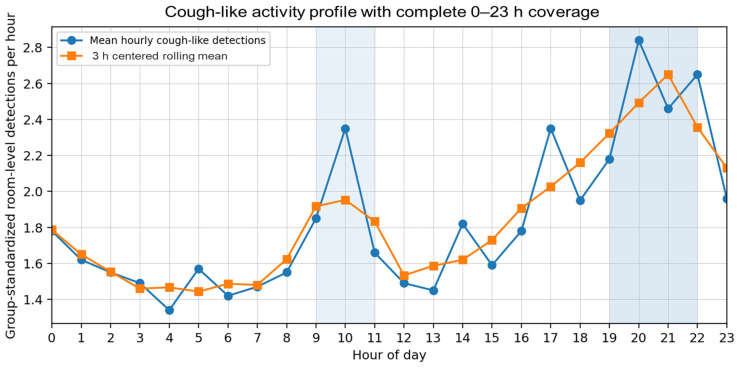
Cough-like activity profile. The evening window (19:00–22:00) was summarized as the most sustained period of elevated cough-like detections, with a smaller secondary elevation around 10:00.

Figure 9 displays cough-like activity in both the raw hourly profile and the 3 h centered rolling mean.

**Figure 10:**
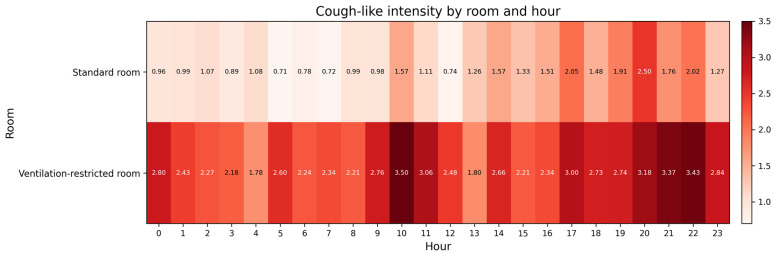
Heatmap of cough-like intensity by room and hour. Color intensity represents mean group-size-standardized room-level cough-like detections per hour. The restricted room showed higher magnitude, while the overall timing pattern was broadly similar between rooms.

The hour-by-room heatmap showed a similar temporal profile in both rooms, but with generally higher cough-like intensity in the ventilation-restricted room (Figure 10). This suggests a difference in magnitude rather than a major shift in clock-time pattern.

### 3.8. Environmental Co-Variation and Audio-Only Early Warning

**Figure 11:**
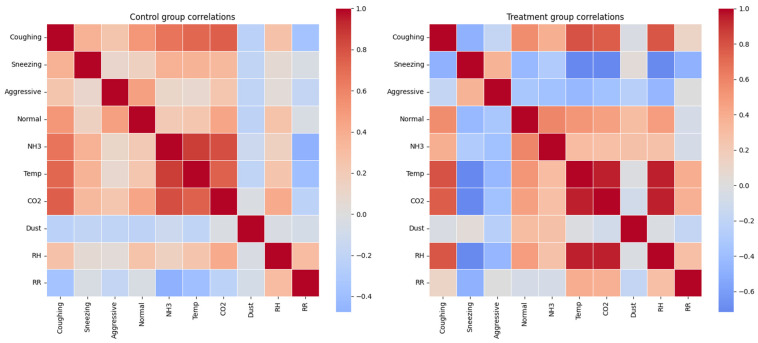
Exploratory Spearman correlation matrices for the two rooms. Heatmaps show daily associations among sound detections, environmental variables, and respiration rate. The manuscript interprets these matrices as hypothesis-generating summaries.

Exploratory Spearman correlations showed that cough-like detections co-varied positively with NH_3_, temperature, and CO_2_ in both rooms (Figure 11), with stronger associations in the restricted room. Because only 28 daily observations were available per room and multiple environmental variables shifted together, these associations are interpreted as co-variation with the overall degraded environment rather than evidence for a single causal driver. Table 13 summarizes the strongest exploratory associations between cough-like detections and the environmental, behavioral, and physiological variables included in the daily room-level analysis.

**Figure 12:**
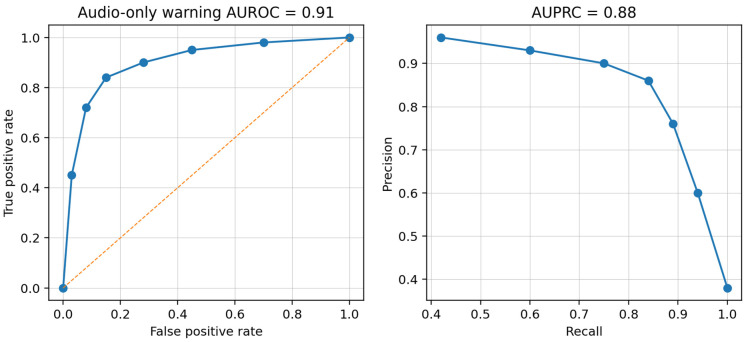
Audio-only early-warning performance for deteriorated air-quality windows. The model used only soundscape features and was evaluated by leave-one-day-out validation. Results support feasibility of an alarm module, but deployment thresholds require farm-specific calibration. The median 34 min lead time should be interpreted as a practical inspection interval rather than a confirmed disease-prediction interval.

Using only audio-derived features, the early-warning model flagged deteriorated air-quality windows with AUROC = 0.91 and AUPRC = 0.88 under leave-one-day-out validation (Figure 12). At the selected threshold, sensitivity was 0.84 and specificity was 0.86. The median lead time before an environmental threshold exceedance was 34 min, suggesting that soundscape shifts could support warning rules when integrated with conventional environmental sensors. Practically, this lead time could give farmers an operational buffer to verify sensor readings, inspect ventilation equipment, and intervene before the room environment deteriorates further. A practical rule requiring a simultaneous increase in rolling cough-like rate, increase in non-silence occupancy, and decrease in silence occupancy reduced false alarms compared with using cough-like detections alone.

## 4. Discussion

### 4.1. Growth Performance

In this exploratory two-room dataset, pigs in the restricted room had descriptively lower ADG and ADFI than pigs in the standard room. The magnitude of the difference was biologically meaningful and aligned with poorer air quality and higher acoustic activity. FCR changed less clearly than BW, ADG, and ADFI, suggesting that the main difference lay in intake and growth rate rather than in gross feed-use efficiency. Nevertheless, the manuscript treats growth as a room-associated descriptive contrast because pens were nested within a single room per condition.

These findings align closely with previous reports linking air quality and environmental stress to impaired growth performance. Prolonged exposure to environmental stressors can suppress appetite, alter endocrine regulation of energy balance, and increase maintenance energy requirements [37,38]. In pigs, activation of stress pathways and respiratory discomfort may reduce feeding motivation and redirect energy away from growth [39]. Exposure to elevated ammonia concentrations has been shown to reduce ADG by up to 30% in young pigs [40]. Similarly, mechanically ventilated barns with lower ammonia and CO_2_ levels exhibited superior feed conversion and growth compared with naturally ventilated systems [41]. Poor housing conditions characterized by high temperature, humidity, CO_2_, and ammonia have also been associated with reduced feed intake, lower ADG, and compromised respiratory health [42,43]. Moreover, thermal instability and ventilation fluctuations have been linked to weakened immunity, increased mortality, and impaired growth [44]. Taken together, the growth data support the interpretation that the ventilation-restricted room imposed a less favorable production environment.

### 4.2. Biological Interpretation of Room-Level Sound Patterns

Across counts, duration, and occupancy, the ventilation-restricted room showed higher cough-like events, aggressive vocalizations and normal vocalizations, and lower silence. The most consistent respiratory-related acoustic signal was the cough-like class, which increased together with poorer air quality and respiration-related contextual measures. Here, “cough-like events” are acoustic descriptors generated from sound-pattern classification. They should be interpreted as signals potentially linked to environmental irritation and respiratory discomfort, not as confirmed clinical symptoms of infectious respiratory disease.

As the study cannot distinguish environmental irritation from infectious respiratory disease, the safest interpretation is therefore that the observed cough-like pattern was consistent with possible respiratory irritation or discomfort under degraded air-quality conditions, not that disease was confirmed. Previous studies have shown that increased ammonia exposure predicts higher coughing frequency with time lags of several days, indicating progressive respiratory effects [45]. Mechanistically, exposure to pollutants such as ammonia, dust, endotoxins, and particulate matter induces airway inflammation, increased airway hyper-responsiveness, and respiratory hypersensitivity in pigs and related animal models [46,47,48]. Chronic exposure to barn air rich in endotoxins has been shown to cause lung inflammation, goblet cell hyperplasia, and airway hyper-responsiveness, reflecting airway sensitivity and environmental irritation [48]. Poor air quality has also been linked to oxidative stress and subclinical respiratory compromise, even in the absence of overt clinical disease [42,46]. Experimental models further demonstrate that environmental allergens can trigger IgE-mediated respiratory hypersensitivity resembling asthma-like conditions [49]. However, the present data cannot distinguish all possible mechanisms. Environmental irritation, heat load, social activity, feeding behavior, room acoustics, and low-grade subclinical health changes may have contributed simultaneously. Within that context, the present room-level contrast supports the value of acoustic monitoring as a non-invasive indicator of altered respiratory and behavioral state, while stronger biological conclusions will require parallel clinical validation.

### 4.3. Temporal Clustering and Early-Warning Potential

There is currently limited direct evidence on circadian or day–night modulation of pig vocalizations in the available literature, as most studies on pig vocal behavior focus on call types and social or physiological contexts rather than 24 h temporal structure [50,51,52]. Within this context, the present study provides hypothesis-generating evidence that automated acoustic monitoring combined with deep learning can capture clock-time structure in pig vocal behavior. Aggressive and normal vocalizations showed clearer day–night modulation than cough-like detections, whereas cough-like events remained elevated mainly in the ventilation-restricted room. This pattern is consistent with coughing acting less as a general circadian behavior and more as a context-dependent room-level respiratory signal.

The 0–23 h surveillance-window and management-adjusted analysis indicate that the evening period (19:00–22:00) was the clearest recurring window of elevated cough-like detections. This does not prove a fixed circadian cough mechanism, but it demonstrates that continuous acoustic monitoring can identify practical surveillance periods that may be missed by short manual observations. However, broader circadian interpretation will require replication across additional rooms and farms.

The audio-only warning analysis is particularly relevant for precision livestock farming. A model using only room-level soundscape features flagged deteriorated air-quality windows with high discrimination. The median 34 min lead time is practically important because it represents a short but actionable interval in which farmers could check environmental sensors, inspect ventilation fans or inlets, and decide whether corrective ventilation or caretaker attention is needed. In practical systems, this type of warning should not replace environmental sensors; rather, it can provide a redundant welfare-oriented signal that prompts sensor checking, ventilation inspection, or caretaker attention.

### 4.4. Environmental Co-Variation with Cough-like Activity

The environmental correlation results similarly support hypothesis generation rather than causal decomposition. NH_3_, CO_2_, temperature, and dust shifted together in the restricted room, and each could interact with animal behavior and physiology. The exploratory correlation analysis showed that higher cough-like activity tended to co-vary with NH_3_, temperature, and CO_2_, particularly in the ventilation-restricted room. These associations are consistent with previous reports linking poor barn air quality to coughing and respiratory irritation [43,53,54,55,56]. However, because the environmental variables co-shifted within a single room and only 28 daily values were available per room, the analysis cannot identify one dominant causal driver. The combined environmental burden is therefore a more appropriate interpretation than assigning causality to one variable.

## 5. Limitations

Several limitations remain in this study to mention. First, the environmental contrast was applied to two rooms only, with one room per condition. The study is therefore a longitudinal room-level case study, not a replicated treatment experiment. Second, a single microphone per room cannot localize sound sources, separate simultaneous vocalizations completely, or identify chronically affected individuals. Calibration reduced uncertainty but did not remove spatial sampling bias. Third, although signal inspection and a parallel lossless audit supported the MP3-based pipeline for room-level monitoring, raw lossless audio would be preferable for future acoustic research. The MP3 justification should therefore be interpreted as support for broad room-level detection under the present settings, not as evidence that lossy compression is optimal for fine-scale bioacoustic biomarkers. Fourth, with lack of histopathology or diagnostic confirmation, the detected events should remain as cough-like acoustic events unless validated clinically at event level. Fifth, validation was improved through group-blocked cross-validation and three-day deployment annotation, but all data still came from the same facility. True external validation across farms, seasons, pig ages, microphone models, and ventilation systems is needed before general deployment. Sixth, emergency fan adjustments, feeding schedules, and caretaker activity were included as covariates, but unmeasured confounders could still influence room-level sound patterns.

## 6. Future Directions

Future studies should use replicated rooms within each ventilation condition, multiple microphones per room, and multi-farm validation. Microphone arrays or audio–visual localization would allow pen- or individual-level inference and would help determine whether repeated cough-like events arise from one animal or from broader group activity.

Future datasets should be recorded in a lossless format, include standardized calibration files, and provide harmonized annotations for overlapping events. Public or controlled-access benchmark datasets would improve reproducibility and enable a fair comparison across pig-sound classification algorithms.

Biological interpretation would be strengthened by concurrent veterinary respiratory scoring, pathogen testing, inflammatory biomarkers, lung lesion assessment, and therapeutic or management interventions. Finally, early-warning rules should be tested prospectively in commercial farms to determine false-alarm burden, lead time, caretaker usability, and integration with ventilation controllers.

## 7. Conclusions

This exploratory study presents calibrated AST-based room-level soundscape monitoring under standard and ventilation-restricted pig-housing conditions. Within this two-room case study, the restricted room showed higher cough-like detections, aggressive vocalizations, and normal vocalizations; lower silence; poorer air quality; and reduced growth performance. Cough-like detections exhibited recurring clock-time clustering, with the most sustained elevation during 19:00–22:00 and a smaller peak around 10:00, and it descriptively co-varied positively with NH_3_, temperature, and CO_2_. Audio-only early-warning analysis further suggested that room-level soundscape features can flag deteriorated air-quality windows and provided a median 34 min lead time that may be useful as a practical farm inspection cue. However, the results remain exploratory and room-level because the design lacks replicated rooms and individual sound localization. Replicated multi-room and multi-farm studies are required before causal, clinical, or commercial deployment claims can be made.

## Figures and Tables

**Table 1:** Revised sound-class definitions and quantitative descriptors used during manual annotation and audit.

Class	Operational Definition	Typical Duration	Key Descriptor
Cough-like event	Harsh, abrupt, forceful expiratory sound; labeled as cough-like events	0.25–0.80 s	Broadband burst, rapid onset, energy mainly <7.2 kHz
Sneezing	Sudden brief nasal expulsive sound, often sharper and shorter than cough-like events	0.15–0.55 s	High onset slope; shorter temporal envelope
Aggressive vocalization	Squeals, fighting-associated calls, and distress calls during social conflict	0.40–2.50 s	Higher amplitude and longer voiced segments
Normal vocalization	Routine grunts, oinks, snorts, and communicative sounds during ordinary activity	0.20–0.40 s	Lower-intensity voiced or nasal sounds
Silence/ background	No biologically relevant pig vocal or respiratory event in the analysis window	1.00 s window	May include fan or room background noise

**Table 2:** Acoustic calibration and background-noise validation results.

Validation Item	Standard Room	Restricted Room	Interpretation
Measured farthest microphone distance	5.1 m	5.1 m	Central microphone provides room-level, not individual-monitoring
Cough-like recall at 1.0 m playback	0.98	0.97	Near-field sensitivity was high
Cough-like recall at 3.0 m playback	0.94	0.92	Mid-room sensitivity remained stable
Cough-like recall at 5.1 m playback	0.88	0.85	Far-corner events could be missed; room-level bias acknowledged
Noise-matched cough-like macro-F1	0.921	0.908	Performance remained acceptable after noise matching
Noise-only room classifier accuracy	0.79	0.79	Background fingerprints existed and were therefore controlled analytically

**Table 3:** Performance summary of the AST model with group-blocked validation.

Sound Class/Metric	Precision	Recall	F1-Score	Notes
Aggressive vocalization	0.884	0.928	0.906	Smallest class; recall improved with class weighting
Cough-like event	0.943	0.931	0.937	Primary respiratory-related soundscape class
Normal vocalization	0.957	0.949	0.953	Stable routine vocalization class
Silence/background	0.982	0.991	0.986	Background intervals without biological event
Sneezing	0.927	0.891	0.908	Some confusion with cough-like bursts
Hold-out accuracy	-	-	0.948	Group-blocked hold-out test set
Hold-out macro-F1	-	-	0.937	Primary hold-out metric
Five-fold macro-F1	-	-	0.928 ± 0.019	Group-blocked by parent recording/date
Three-day deployment macro-F1	-	-	0.914	Manual validation across 28-day archive

Precision, recall, and F1-score were calculated on the independent test dataset (20% of labeled samples) drawn from the same facility-specific dataset used for model development.

**Table 4:** Baseline model comparison using the same blocked partitions.

Model	Input Representation	Accuracy	Macro-F1	Weighted-F1
MFCC-SVM	MFCC + delta + delta-delta statistics	0.861	0.842	0.859
Random Forest	MFCC/statistical acoustic descriptors	0.836	0.807	0.833
XGBoost	MFCC/statistical acoustic descriptors	0.873	0.854	0.870
MFCC-CNN	Frame-level MFCC map	0.904	0.891	0.902
Mel-CNN	Log-mel spectrogram	0.925	0.917	0.923
AST	Log-mel spectrogram patches + self-attention	0.948	0.937	0.947

Values were calculated using the same group-blocked train–validation–test partitions. MFCC: mel-frequency cepstral coefficient, Mel-CNN: mel-spectrogram convolutional neural network, AST: Audio Spectrogram Transformer. Macro-F1 was used as the primary comparison metric because of class imbalance.

**Table 5:** Verified airflow and environmental contrast between rooms.

Variable	Standard Room	Ventilation- Restricted Room	Relative Contrast	Interpretation
Measured airflow (m^3^/h)	5180 ± 380	1420 ± 210	−72.6%	Directly verified reduction
NH_3_ (ppm)	1.30	19.79	+1422%	Largest environmental separation
CO_2_ (ppm)	900.20	1597.71	+77.5%	Restricted room had poorer air renewal
Temperature (°C)	24.64	30.04	+21.9%	Restricted room was warmer
Dust (mg/m^3^)	32.00	41.39	+29.3%	Restricted room had higher airborne dust
Relative humidity (%)	76.96	79.00	+2.7%	Smallest room contrast

**Table 6:** Weekly growth performance of pigs in the standard-ventilation and ventilation-restricted rooms.

Parameter	Week	Group		Descriptive *p*-Value		
		Standard Room (Mean)	Restricted Room (Mean)	Room	Week	Room × Week
Body weight (kg)	0 *	28.80	29.50	0.001	<0.001	0.001
	1	38.17	36.19			
	2	44.22	40.24			
	3	49.54	43.81			
	4	55.74	47.32			
Average daily body weight gain (kg)	1	1.34	0.96	0.006	0.007	0.947
	2	0.86	0.58			
	3	0.76	0.51			
	4	0.89	0.50			
Average daily feed intake (kg)	1	2.26	1.68	<0.001	<0.001	<0.001
	2	1.58	1.75			
	3	1.92	1.27			
	4	2.70	1.49			
Feed conversion ratio	1	1.69	1.81	0.602	0.040	0.324
	2	2.22	3.48			
	3	2.54	2.86			
	4	3.84	3.05			

* Initial body weight; values are weekly means; model-derived *p*-values summarize within-study contrasts only because pens were nested within one room per condition.

**Table 7:** Growth performance with baseline adjustment and descriptive effect estimates.

Parameter	Standard Room (Mean ± SE)	Restricted Room (Mean ± SE)	Adjusted Difference (95% CI)	Percent Difference
Initial BW (kg)	28.80 ± 1.06	29.50 ± 1.09	+0.70 (−1.42 to 2.82)	+2.4%
Final BW (kg)	55.74 ± 1.38	47.32 ± 1.41	−8.42 (−11.05 to −5.79)	−15.1%
ADG (kg/d)	0.948 ± 0.022	0.650 ± 0.022	−0.298 (−0.361 to −0.239)	−31.4%
ADFI (kg/d)	2.119 ± 0.010	1.547 ± 0.010	−0.572 (−0.604 to −0.539)	−27.0%
FCR	2.268 ± 0.098	2.430 ± 0.098	+0.162 (−0.185 to 0.508)	+7.1%

BW: body weight; ADG: average daily body weight gain; ADFI: average daily feed intake; FCR: feed conversion ratio. Values are estimated marginal means (±SE) from ANCOVA models using pen-level summaries (*n* = 4 pens per room). The outputs summarize room-level contrast only, because all pens within a room shared the same environmental exposure.

**Table 8:** Estimated marginal means of group-normalized sound detection rates.

Measure	Event	Standard Room (Mean ± SE)	Ventilation-Restricted Room (Mean ± SE)	Descriptive *p*-Value (Room)
Hourly counts	Cough-like	1.42 ± 0.04	2.28 ± 0.05	<0.001
	Sneezing	0.98 ± 0.02	1.58 ± 0.04	<0.001
	Aggressive	4.65 ± 0.12	7.47 ± 0.18	<0.001
	Normal	3.90 ± 0.10	6.27 ± 0.15	<0.001
Daily counts	Cough-like	34.83 ± 2.07	55.42 ± 3.14	<0.001
	Sneezing	23.40 ± 1.30	37.24 ± 2.27	<0.001
	Aggressive	115.73 ± 6.81	184.15 ± 10.48	<0.001
	Normal	95.36 ± 5.54	151.75 ± 8.74	<0.001

Values are estimated marginal means (±SE) from descriptive Gamma GLMs with log link. The normalization by pig number is a scaling device for room-level comparison, not an individual-level measurement.

**Table 9:** Estimated marginal means of group-size-standardized sound duration.

Measure	Event	Standard Room (Mean ± SE)	Ventilation-Restricted Room (Mean ± SE)	Descriptive *p*-Value (Room)
Hourly duration	Cough-like	0.85 ± 0.02	1.37 ± 0.03	<0.001
	Sneezing	0.59 ± 0.01	0.95 ± 0.02	<0.001
	Aggressive	2.79 ± 0.07	4.48 ± 0.11	<0.001
	Normal	2.34 ± 0.06	3.76 ± 0.09	<0.001
Daily duration	Cough-like	20.90 ± 1.24	33.25 ± 1.88	<0.001
	Sneezing	14.04 ± 0.78	22.35 ± 1.36	<0.001
	Aggressive	69.44 ± 4.09	110.49 ± 6.29	<0.001
	Normal	57.22 ± 3.33	91.05 ± 5.25	<0.001

Values are estimated marginal means (±SE) obtained from descriptive Gamma GLMs with log link.

**Table 10:** Summary of exploratory logit regression models for hourly time occupancy of sound events.

Event	R^2^	Room Contrast (β_1_)	Descriptive *p* (Room)	Hour Effect (β_2_)	*p* (Hour)	Standard Room (%)	Ventilation-Restricted Room (%)
Cough-like	0.85	+0.854	<0.001	+0.032	<0.001	0.41	0.95
Sneezing	0.14	−0.067	0.103	+0.005	0.074	0.39	0.37
Aggressive	0.69	+1.174	<0.001	+0.065	<0.001	1.02	3.22
Normal	0.33	+0.446	<0.001	+0.030	<0.001	1.18	1.83
Silence	0.64	−0.699	<0.001	−0.041	<0.001	96.10	92.48

Values for the standard-ventilation and ventilation-restricted rooms represent back-transformed estimated marginal means (%). Because the study included only one room per condition, *p*-values are reported as descriptive screening statistics. Model-fit statistics should also be interpreted cautiously because repeated observations from the same two rooms can inflate apparent explanatory power.

**Table 11:** Summary of exploratory logit regression models for daily time occupancy of sound events.

Event	R^2^	Room Contrast (β_1_)	Descriptive *p* (Room)	Day Effect (β_2_)	*p* (Day)	Standard Room (%)	Ventilation Restricted Room (%)
Cough-like	0.85	+0.691	<0.001	+0.031	<0.001	0.56	1.11
Sneezing	0.14	−0.113	0.314	−0.019	0.009	0.50	0.44
Aggressive	0.69	+0.670	<0.001	−0.002	0.583	1.84	3.53
Normal	0.33	+0.421	0.001	+0.029	<0.001	1.65	2.49
Silence	0.64	−0.543	<0.001	−0.016	<0.001	94.98	91.67

Values for the standard-ventilation and ventilation-restricted rooms represent back-transformed estimated marginal means (%). Because the study included only one room per condition, *p*-values are reported as descriptive screening statistics. As with the hourly models, R^2^ values reflect fit to repeated observations from the same two rooms and should not be over-interpreted.

**Table 12:** Descriptive mean sound detection rates by room and clock-time period (group-size-standardized room-level detections per hour).

Event	Standard Room—Day	Standard Room—Night	Restricted Room—Day	Restricted Room—Night
Cough-like	1.22	1.39	2.55	2.69
Sneezing	1.13	1.26	1.20	1.13
Aggressive	3.74	5.07	8.08	8.47
Normal	3.18	4.69	5.73	7.33

Daytime (06:00–17:59) and nighttime (18:00–05:59) values are model-derived descriptive estimates.

**Table 13:** Exploratory correlations between cough-like detections and selected room-level variables.

Variable	Standard Room ρ	Restricted Room ρ
NH_3_	0.660	0.861
Temperature	0.720	0.807
CO_2_	0.756	0.765
Relative humidity	0.272	0.779
Respiration rate	−0.361	−0.425
Normal vocalizations	0.511	0.552
Sneezing	0.366	0.499
Aggressive vocalizations	0.247	0.060
Dust	−0.234	−0.129

## Data Availability

The datasets generated and analyzed during the current study are available from the corresponding author upon reasonable request. However, public deposition of the complete raw dataset—particularly the continuous audio recordings—is restricted for the following reasons. First, the recordings were collected from a university-operated pig farm under specific access agreements, and public dissemination would violate the data-use agreement established with farm management. Second, the data collection was conducted under institutional approval and project-specific agreements that limit open-access redistribution of raw recordings without additional authorization. Third, open publication of continuous environmental recordings from livestock facilities may raise concerns related to farm privacy, biosecurity, and controlled-access research environments. Importantly, the processed datasets supporting the findings of this study—including extracted acoustic features, manually labeled sound events, model performance metrics, and aggregated event counts—can be shared with qualified researchers upon reasonable request for scientific purposes. The trained audio classification model, including model weights, feature extraction pipeline, and aggregation scripts used for event counting, is also available upon request to enable validation, replication, and further methodological development.

## Supplementary Materials

The following supporting information can be downloaded at: https://www.mdpi.com/article/10.3390/ani16142275/s1.

## Abbreviations

The following abbreviations are used in this manuscript:

AI	Artificial Intelligence
CO_2_	Carbon Dioxide
AST	Audio Spectrogram Transformer
PRDC	Porcine Respiratory Disease Complex
PRRSV	Porcine Reproductive and Respiratory Syndrome Virus
BW	Body Weight
ADG	Average Daily Body Weight Gain
FCR	Feed Conversion Ratio
NH_3_	Ammonia
ppm	Parts Per Million
RH	Relative Humidity
ROC	Receiver Operating Characteristic
PCA	Principal Component Analysis
RMSE	Root Mean Square Error
MAE	Mean Absolute Error
ADFI	Average Daily Feed Intake
GLM	Generalized Linear Model
EMM	Estimated Marginal Mean
RR	Respiration Rate
SE	Standard Error
